# Reliability of Clinician Rated Physical Effort Determination During Functional Capacity Evaluation in Patients with Chronic Musculoskeletal Pain

**DOI:** 10.1007/s10926-013-9470-9

**Published:** 2013-08-22

**Authors:** M. A. Trippolini, P. U. Dijkstra, B. Jansen, P. Oesch, J. H. B. Geertzen, M. F. Reneman

**Affiliations:** 1 Department of Work Rehabilitation, Rehaklinik Bellikon, Suva Care, 5454 Bellikon, Switzerland; 2 Department of Rehabilitation Medicine, Center for Rehabilitation, University Medical Center Groningen, University of Groningen, Groningen, The Netherlands; 3 Department of Oral and Maxillofacial Surgery, University Medical Center Groningen, University of Groningen, Groningen, The Netherlands; 4 Research Department, Rehabilitation Centre Valens, Valens, Switzerland; 5 Department of Rheumatology, Rehabilitation Centre Valens, Valens, Switzerland

**Keywords:** Rehabilitation, Pain, Disability evaluation, Lifting, Work capacity evaluation

## Abstract

*Introduction* Functional capacity evaluation (FCE) can be used to make clinical decisions regarding fitness-for-work. During FCE the evaluator attempts to assess the amount of physical effort of the patient. The aim of this study is to analyze the reliability of physical effort determination using observational criteria during FCE. *Methods* Twenty-one raters assessed physical effort in 18 video-recorded FCE tests independently on two occasions, 10 months apart. Physical effort was rated on a categorical four-point physical effort determination scale (P_ED_) based on the Isernhagen criteria, and a dichotomous submaximal effort determination scale (S_ED_). Cohen’s Kappa, squared weighted Kappa and % agreement were calculated. *Results* Kappa values for intra-rater reliability of P_ED_ and S_ED_ for all FCE tests were 0.49 and 0.68 respectively. Kappa values for inter-rater reliability of P_ED_ for all FCE tests in the first and the second session were 0.51, and 0.72, and for S_ED_ Kappa values were 0.68 and 0.77 respectively. The inter-rater reliability of P_ED_ ranged from κ = 0.02 to κ = 0.99 between FCE tests. Acceptable reliability scores (κ > 0.60, agreement ≥80 %) for each FCE test were observed in 38 % of scores for P_ED_ and 67 % for S_ED_. On average material handling tests had a higher reliability than postural tolerance and ambulatory tests. *Conclusion* Dichotomous ratings of submaximal effort are more reliable than categorical criteria to determine physical effort in FCE tests. Regular education and training may improve the reliability of observational criteria for effort determination.

## Introduction

Individuals suffering from chronic nonspecific musculoskeletal pain (CMP) such as back and neck pain are often restricted in performing activities of daily living and work [1, 2]. The financial burden of CMP on society arises mainly due to indirect costs because of temporary or permanent work disability. Work disability due to CMP may be associated with reduced activity levels and work performance [3, 4]. Functional capacity evaluation (FCE) in addition to self-reported measures have been recommended for a comprehensive assessment of physical work performance for persons with CMP [5–8].

Functional capacity evaluation employs physical performance tests such as lifting, postural tolerance tests, repetitive movements, and ambulation to assess work-related functioning [9]. Discrepancies in FCE outcomes and the physical workload of a patient may be addressed in rehabilitation to restore this imbalance [10–12]. Moreover, FCEs are used to evaluate the effects of rehabilitation and determine fitness-for-work, and as such FCEs may facilitate the return-to-work process or prelude case closure [13–17].

To determine physical capacity during the FCE the patient must perform to his or her maximum level of physical ability. The level of physical effort during FCE is estimated by the evaluator, based on observational criteria during material and non-material handling tests [9, 18]. Submaximal effort is assumed when a person stops a FCE test before the criteria indicative of maximal effort are observed. Because clinical decision-making is based on the results of FCE, sound clinimetric properties of observational criteria are required to determine physical effort. Acceptable reliability of physical effort determination FCE tests such as lifting has been reported [19, 20]. However, the reliability of non-material handling tests such as kneeling and forward bending has rarely been studied [21–25]. Moreover, most studies on lifting tests were performed by FCE experts, which limits the generalizability and applicability of the study results among less experienced raters [25–27].

The aim of this study was to determine the intra- and inter-rater reliability of physical effort determination of FCE tests in patients with CMP. A second aim was to investigate whether an increase in rater experience would alter the reliability of physical effort determination.

## Methods

### Procedures, Patients and Video Sequences

Video tape-recordings were taken during FCEs, performed in a work rehabilitation setting. FCE tests were performed according to the Isernhagen test procedure, which claims to measure a person’s physical capacity to safely engage in work-related activity [28]. Four patients (3 with non-specific low back pain and 1 with non-specific neck pain, mean age 35.5 years, range 21–49 years) were recruited based on convenience. All patients were instructed how to perform the test, and that they were expected to perform maximally. Testing could be terminated for four reasons: the participant stopped because of, for example, pain; the observer deemed testing to have become over safe maximum based on criteria for effort determination (Appendices 1, 2); heart rate exceeded 85 % of the age-related maximum (220 minus age of participant); or a predefined time limit was reached. All patients gave written consent to be video-recorded. Eighteen videos from 11 FCE tests with a total duration of 28 min were selected. The videos were mute recorded. For each test information was provided on a standardized form regarding heart rate at the beginning and end of the test, and weight lifted in kilograms (for material handling tests) or duration (for static posture, or walking, stair climbing).

### Raters

A convenience sample of 21 physiotherapists (11 female, 10 male) from Bellikon rehabilitation clinic (Switzerland) served as a representative sample of raters. Nineteen had attended the official 2-day FCE training course provided by the Swiss Rehabilitation Association [18]. Prior to the study all had performed at least ten 1-day FCEs in the previous year [median 30, interquartile range (IQR) 20–33] and had a minimum of 1 year work experience in work rehabilitation (median 3, IQR 2–3), and a minimum professional practice experience of 1 year (median 5 years, IQR 3–12.5).

### Physical Effort Determination During FCE Tests

The 18 videos were shown in a classroom to all the raters at the same time. Prior to the showing the raters were instructed about the procedure of the rating. The ratings of physical effort were filled in a standardized form with a pencil. The videos consisted of 18 tests. When a test was finished and all participants had rated that test, then the next test was shown. Raters were not allowed rewind the video or to stop a video while a test was shown. Each video was shown once per session. Raters were blinded each other’s ratings. Each video was rated according to observational criteria indicative of physical effort for material handling tests as “light to moderate”, “heavy” or “maximal” (Appendix 1). Observational criteria for postural tolerance tests and ambulation tests were rated on a scale from “No or slight functional problem/limitation”, “some functional problem/limitation” to “substantial functional problem/limitation” (Appendix 2). This categorical scale was termed physical effort determination (P_ED_) scale. If a test was performed unsafely it was classified as “over safe maximum”, when observed performance exceeded the maximum observational criteria for physical effort level during work-related tasks (Appendices 1, 2). Tests were scored as “not classifiable” when the patient interrupted the FCE test at the very start or the observed effort was not clearly interpretable to the raters and no conclusions could be drawn. Submaximal effort was assumed when a patient stopped a material or non-material handling test before the FCE rater observed sufficient criteria indicative of maximal weight, or significant functional problems/limitation as described in Appendices 1 and 2. This dichotomous scale was termed submaximal effort determination (S_ED_).

Maximal effort was defined as the highest safe ability of a person during a FCE test [9]. An FCE was considered safe when no formal complaints of injury or serious adverse effects were filed by the patients, and when increased symptoms returned to or below their pre-FCE level [29].

The observers rated each video twice, in September 2010 (session 1) and in July 2011 (session 2). Between these sessions each rater performed approximately 30 short FCEs (material handling tests only), as part of the regular clinical procedure of a work rehabilitation program. All raters attended both sessions. Data extraction into the database was performed by an individual who was not involved in the data analysis.

Both patients and raters agreed that their data would be used either for the scope of research or education. Because this study was part a regular educational video based training, no ethical approval was required. However, this study was part of a research project approved by the Medical Ethics Committee of Canton Aargau, Switzerland (EK AG 2010/055) [30].

### Data Analysis

Intra-rater reliability was assessed by comparing the scores from the first rating session with the scores from the second session for each rater. Inter-rater reliability was assessed twice: by comparing the scores between all the raters in session 1 and 2. Category 5 “not classifiable” was excluded from the analyses. Inter-rater and intra-rater reliability was calculated using Cohen’s Kappa values for dichotomous data, and squared weighted Kappa values for categorical data and percentages of agreement. A percentage of agreement of 80 % or more was judged as acceptable. If agreement was ≥80 % and Kappa was κ > 0.60 then reliability values were considered as acceptable [31]. AGREE (Agree, Version 7.002) was used to analyze Kappa for multiple observer categories [32] and the ONLINE KAPPA CALCULATOR was used for multiple raters [33]. All other analyses were performed using SPSS (Statistical Package for Social Sciences, Version 20, 2011).

## Results

### Intra-rater Reliability of Physical Effort Determination for all FCE Tests

Excluding category 5 “not classifiable” resulted in 325 ratings for the categorical scale for physical effort determination (P_ED_) (Table 1) and 376 ratings were performed for the dichotomous scale for submaximal effort (S_ED_) (Table 2).

**Table 1:** Cross tabulation of the categorical ratings for physical effort determination (P_ED_) in session 1 and 2

	Category^a^	Description	Session 2					Total
			1	2	3	4	5	
Session 1	1	Light to medium effort	156	32	2	1	4	195
	2	Heavy effort	40	70	5	1	5	121
	3	Maximum effort	2	8	5	0	8	23
	4	Over safe maximum	0	3	0	0	0	3
	5	Not classifiable^b^	7	2	0	0	27	36
Total			205	115	12	2	44	378

^a^Categories 1–5 are described in Appendices 1 and 2; ^b^Category 5 “not classifiable” was excluded from the analyses

**Table 2:** Cross tabulation of the categorical ratings for submaximal effort determination scale (S_ED_) in session 1 and 2

	Category^b^	Session 2		Total
		Criteria for maximal physical effort observed^a^		
		Yes	No	
Session 1	Yes	241	27	268
	No	23	85	108
Total		264	112	376

^a^Yes = observed effort was assumed to be indicative for maximal effort as described in Appendices 1 and 2 when patient performed the material or non-material handling test. ^b ^No = Submaximal effort was assumed when a patient stopped a material or non-material handling test *before* the FCE rater observed sufficient criteria indicative of maximal weight, or significant functional problems/limitation as described in Appendices 1 and 2

### Reliability of Physical Effort Determination (P_ED_)

The intra-rater reliability of P_ED_ for all FCE tests in both sessions together was κ = 0.49 (95 % CI 0.22–0.75). The inter-rater agreement of P_ED_ for all FCE tests increased from 73 % (session 1) to 85 % (session 2). Kappa values as a measure of inter-rater reliability of P_ED_ for all FCE tests increased from session 1 (0.51; 95 % CI 0.23–0.80) to session 2 (0.72; 95 % CI 0.49–0.94). Mean Kappa values for inter-rater reliability of P_ED_ increased from session 1 to 2 for material handling (0.17), postural tolerance (0.21) and ambulation (0.03) (Table 3). Mean agreement values of material handling, postural tolerance and ambulation tests ranged from 54 to 75 % for inter- and intra-rater reliability (Table 3).

**Table 3:** Inter- and intra-rater reliability for each FCE test

Category	Test (n)	Physical effort determination scale (P_ED_)^a^						Submaximal effort scale (S_ED_)^b^					
			Inter		Inter	Intra			Inter		Inter	Intra	
		Session 1	Session 1	Session 2	Session 2	Session 1–2		Session 1	Session 1	Session 2	Session 2	Session 1–2	
		%	κ	%	κ	%	κ	%	κ	%	κ	%	κ
M	One-handed carrying (4)	68	0.57	*80*	*0.74*	71	0.54	75	0.49	75	0.49	76	0.29
M	Lifting floor to waist (4)	58	0.43	73	*0.64*	67	0.47	*85*	*0.70*	*88*	*0.76*	*85*	0.05
M	Two-handed horizontal lift (2)	50	0.34	47	0.29	66	0.34	*95*	*0.90*	*100*	*1.00*	*100*	*1.00*
M	Lifting waist to overhead (2)	66	0.55	*91*	*0.88*	*81*	0.60	*100*	*1.00*	*100*	*1.00*	*100*	*1.00*
Mean		61	0.47	73	0.64	71	0.49	89	0.77	91	0.81	90	0.59
P	Kneeling (1)	*80*	*0.73*	*90*	*0.99*	*84*	−0.08	68	0.35	*100*	*1.00*	*100*	*1.00*
P	Forward bend sitting (1)	44	0.25	33	0.11	55	NA	68	0.35	*90*	*0.81*	76	−0.08
P	Overhead working (1)	42	0.22	79	*0.72*	50	0.35	74	0.49	*90*	*0.81*	*80*	−0.08
Mean		55	0.40	67	0.61	63	0.14	70	0.40	93	0.87	85	0.28
A	Stair climbing (1)^c^	62	0.49	*100*	*1.00*	76	0.00	*90*	*0.80*	*100*	*1.00*	*100*	*1.00*
A	Stair climbing (1)^d^	27	0.02	0	−0.33	74	NA	*100*	*1.00*	*100*	*1.00*	*100*	*1.00*
A	Walking (1)	73	*0.64*	68	0.57	75	0.14	56	0.12	57	0.14	*90*	*0.76*
Mean		54	0.38	56	0.41	75	0.07	82	0.64	86	0.71	97	0.92

Inter: inter-rater reliability; intra: intra-rater reliability; %: percentage agreement; κ: Cohen’s Kappa values for dichotomous, Squared weighted Kappa for categorical data; ^a ^observational criteria for determination of physical effort during material and non-material handling tests (see Appendices 1, 2); ^b^ submaximal effort was assumed, when a participant stopped a material or non-material handling tests before the FCE rater observed sufficient observational criteria indicative of maximal effort; M: material handling tests; P: postural tolerance tests; A: ambulation tests; (n): number of videos; ^c^ short video length until patient stops; ^d^ full video length of the test 10 × 10 stairs up and down; NA: not applicable, due to lack of cell filling. Italicised values criteria for acceptable reliability (agreement ≥80 %, κ > 0.60)

### Reliability of Submaximal Effort Determination (S_ED_)

For S_ED_ the intra-rater reliability for all FCE tests in both sessions together was κ = 0.68 (95 % CI 0.60–0.76). Kappa values as a measure of inter-rater reliability of S_ED_ for all FCE tests increased from session 1 (0.68; 95 % CI 0.60–0.76) to session 2 (0.77; 95 % CI 0.70–0.84). Mean Kappa values for inter-rater reliability of S_ED_ increased from session 1 to 2 for material handling (0.04), postural tolerance (0.47) and ambulation (0.07) (Table 3). Mean agreement values of material handling, postural tolerance and ambulation tests ranged from 70 to 97 % for inter- and intra-rater reliability (Table 3).

### Comparison Reliability of P_ED_ and S_ED_

In 6 out of 10 tests inter-rater agreement and Kappa values for the P_ED_ were equal or increased from session 1 to session 2. For S_ED_ inter-rater agreement and Kappa values were similar or increased for all 10 tests. The general reliability of S_ED_ was higher than that of P_ED_. The inter-rater reliability (% agreement) of S_ED_ was higher in 8 tests (out of 10) for session 1, and in 8 tests (out of 10) for session 2 than that of P_ED_. The inter-rater reliability (Kappa) of S_ED_ was higher in 7 tests (out of 10) for session 1, and in 8 tests (out of 10) for session 2 than that of P_ED_. For intra-rater reliability (% agreement/Kappa) S_ED_ was higher than P_ED_ in 10 out of 10 and 5 out of 10 tests respectively.

When applying cut-off scores for acceptable reliability (agreement levels ≥80 %, κ > 0.60), 46 % (55 out of 120) of the reliability values fulfilled this criterion (see italicised values in Table 3).

## Discussion

When applying cut-off scores of agreement ≥80 %, κ > 0.60, the overall reliability of P_ED_ and S_ED_ was acceptable for less than half (46 %) of all FCE observations. For S_ED_ reliability was acceptable in the majority (67 %) of the FCE tests. However, the reliability of the P_ED_ was acceptable in only 38 % of tests. Inter- and intra-rater reliability between each FCE test varied considerably. The increase in mean reliability scores from session 1 to session 2 was on average higher in the P_ED_ than in the S_ED_.

S_ED_ during FCE tests can be reliably detected in the majority of cases. However the results of this study are disappointing, as raters reached the required reliability cut-off values for both the P_ED_ and S_ED_ in less than half of the observations. This finding has clinical relevance for four reasons. First: some FCEs claim to support fitness-for-work determination with an extrapolation of FCE results to job demands [14, 34]. The job demands and their frequencies during a working day (occasional, 1–33 %; frequent, 34–66 %; constant 67–100 %) are matched to P_ED_ “maximum”, “heavy” and “light to moderate”. Good reliability of P_ED_ is needed to enable adequate matching between FCE performance and work demands. Second: FCEs have been reported to accurately describe physical capacity only if a person exerts “maximal” voluntary effort [23, 35]. Good reliability of determination of effort is a prerequisite for such a clinical interpretation. Third: FCE reports are used by third parties to inform on the progress of insurance claims. Some interpret submaximal physical effort as ‘unmotivated’. The debate over whether this interpretation is valid is beyond the scope of this paper, but it highlights the relevance of the psychometric properties of this determination. Fourth: whether the FCE score represents maximal or submaximal capacity, and the reasons for performing submaximally, are relevant for designing individualized vocational rehabilitation aimed at improvement of functional capacity.

Compared to three previous reliability studies on material handling tests, our values are clearly lower [22, 23, 26]. In some of these previous studies with high reliability values two-point scales for determination of physical effort were used, which increases the a priori probability for agreement compared to a multiple item scale as in our study. In our study agreement on the dichotomous scale (submaximal effort determination) was substantially higher too. Moreover the results show on average an increase in the agreement and reliability rating on both the P_ED_ and S_ED_ scales when administered 10 months apart, indicating a “learning” effect. Our data support the assumption that postural tolerance tests may be difficult to rate using the FCE observational methods, but that experience can substantially improve reliability. The average agreement and Kappa values for the inter-rater reliability of P_ED_ increased by 0.40 during the 10-month period. This may be partly attributed to experience. The raters participating in this study used 1-day FCEs for the standard assessment of most in-patients. In addition they received one-to-one supervision from an FCE expert once a year, and their superiors supervised each FCE report as part of regular quality control. Based on the observation in this study that experience and basic training increased reliability scores, we suggest that novice raters using the observational criteria are supervised more intensively than in our study. To what extent observational criteria for effort determination can be improved by additional training remains unknown.

The only slight increase in the agreement and reliability of S_ED_ might be due to the high scores obtained in the first observation session. When tests were grouped according to type of task the reliability of the physical effort determination scale was generally lower when applied to postural tolerance tests, such as overhead working and kneeling, than when used with material handling tests. This is consistent with results from studies reporting on forward bend, standing and crouching [25, 35, 36]. Moreover observational criteria seem to be less reliable when applied to ambulation tests such as walking and stair climbing compared to material handling tests [25, 36]. However, the results may be influenced by the fact that postural tolerance tests were not part of the regular 1-day FCE utilized in most in-patients, but were only used when indicated. Thus, raters collected more test-experience with the observation of material handling tests than with postural tolerance. Other possible reasons for the lower reliability of the postural tolerance and ambulation tests could be the ceiling effect due to the predefined maximal time limit of the test or the muscular use at submaximal rates. It is theoretically infeasible to judge maximum effort level when submaximal muscular effort is requested e.g. in the overhead work test, the duration of 5 min is not the requested maximum performance, but a time limit. The results of this study underscore this problem. We suggest that observational criteria of physical effort in postural tolerance and ambulation tests need further refinement. To our knowledge no study has been conducted to determine the validity of observational criteria for postural tolerance and ambulation tests in FCE.

In two videos in which a patient performed the one-handed carrying test, ratings showed low agreement. After rating, we discussed these two videos with the raters and asked them where the difficulty lay. Almost half of the raters responded that these were debatable videos due to the pain behavior of the patient. The maximum performance of a patient is determined by the individuals’ ability, motivation, and other psychosocial factors [37, 38]. However, physical effort determination cannot be used interchangeably with non-organic signs described by Waddell et al., despite some important overlap of the two measurement methods [38]. It has been questioned whether lay persons and health care providers can accurately classify effort during a lifting task performed by actors [39]. Similarly to our results this underscores the challenge of determining effort using a categorical rating scale.

### Strengths and Weaknesses of the Study

The strengths of the study were that the inter- and intra-rater reliability measures were based on the results of a large sample of raters, and multiple observations on patient videos. Compared to most other studies on the reliability of P_ED_, additionally to the material handling tests, we included postural tolerance and ambulatory tests. Furthermore this is to our knowledge the first study on the reliability of observational criteria used in FCE tests based on two ratings taken within a period of 10 months, excluding the risk of recall bias. We used 18 videos instead of real patients to test the reliability of the observers. The results may therefore only partly reflect a FCE performed live with the patient. One may argue that several clinical parameters may not have been visible on video tape, such as respiration, and that the raters did not benefit from three-dimensional vision. Observing videos without sound and communication is relevantly different from a clinical setting. In clinical practice FCE raters observe the same patient at different levels of effort when performing the same FCE test. This might facilitate comparison of their own ratings with their previous observations. Studies should be performed to analyze whether the availability of additional information would have changed the results. This study was performed with a sample of four patients. We might therefore not have seen all types of movement patterns of patients with back pain. Because the study was designed to measure the reliability of the raters observing the performance rather than the reliability of that performance, this may have been adequate. The Kappa statistic has an advantage over percentage of agreement because it corrects for chance [31]. In some tests high agreement between raters was observed and Kappa values were in some cases extremely low. This phenomenon may occur when the variation in row and column totals is low [40]. Furthermore it may be debatable if the cut-off score for Kappa values of κ > 0.60 for acceptable reliability used in our study is enough rigorous when one has to make decisions at the individual patient level [41]. The results should therefore be interpreted accordingly. Category 5, “not classifiable”, was excluded from the analysis for two reasons. First “not classifiable” relates to another dimension than those categories related to effort. Therefore it cannot be analyzed in the effort domain. Secondly, only a few ratings were “not classifiable”, indicating its minor influence.

### Future Studies

Although there have been some advances in the study of reliability of physical effort determination, major gaps remain: for example, what are valid and practical reference standards for determining maximal physical effort during FCE tests? While some experimental studies measuring muscle activity measurements such as surface EMG, superimposed electrical stimulation, and lactate concentration have been performed, they lack practicality for clinical use [42, 43]. How should evidence-based cut-off scores of reliability be defined that are useful for the various purposes of FCE? Future studies should address these unresolved questions and promote the development of a reliable tool for the determination of physical effort, above all for postural tolerance tests.

## Conclusions

The reliability of observing physical effort varied substantially between FCE tests, ranging from unacceptable to good. The dichotomous rating of sub-maximal effort was more reliable than the categorical rating for physical effort determination. However, with both rating scales acceptable reliability values were reached on average only in every second observation, which limits their utility for clinical decision-making. Regular education and training may improve the reliability of observational criteria for effort determination. Further research is needed to develop reliable observation scales.

## Appendix 1

See Table 4.

**Table 4:** Observational criteria for determination of physical effort during material handling tests

Criteria	Light to moderate	Heavy	Maximum
Muscle recruitment			
Prime movers	Normal recruitment	Bulging	Bulging
Accessory muscles	No or only slight muscle recruitment	Distinct recruitment	Bulging
Base of support	Natural stance	Distinctly increased	Very wide base
Posture	No or only slight counterbalance in extension	Distinctly increased counterbalance	Substantial counterbalance
Heart rate and respiration	No or minimal increases in heart rate and respiration	Distinct increases in heart rate and respiration	Substantial increases in heart rate and respiration
Control and safety	Smooth movements	Increasingly controlled movement; might begin to use momentum; execution with difficulty but not yet at the limit	Still safe but unable to maintain control with the addition of any more weight
Pace	Moderate/comfortable pace	Distinctly slower; very deliberate movements	Very slow (an increased pace would affect stability and control)

The level of physical effort during material handling tests was determined on the basis of observational criteria indicative of light to moderate, heavy, or maximal weight load [9, 18, 44]. Maximal effort was assumed when, on the basis of the expertise of the functional capacity evaluation (FCE) rater, sufficient criteria indicative of safe maximal weight were observed. Submaximal effort was assumed when a participant stopped a material handling test before the FCE assessor observed sufficient criteria indicative of maximal weight. Appendix 1 is used with permission from Verein IG Ergonomie, Swiss Association of Rehabilitation

## Appendix 2

See Table 5.

**Table 5:** Observational criteria for determination of physical effort during non-material handling tests

Criteria	No or slight functional problem/limitation	Some functional problem/limitation	Substantial functional problem/limitation
Posture	Maintains normal posture, or slight deviation in posture^a^	Some deviation from normal posture^a^, occasional change of position	Substantial deviation from normal posture^a^, substantial unrest (frequent change of posture position)
Movement pattern	Normal movement pattern, slight deviation from normal^a^, smooth movements or slight muscle stiffness, normal to slightly slower performance	Some deviation from the normal movement pattern^a^, tense movements, markedly slower performance	Substantial deviation from the normal movement pattern^a^, very tense movements, very slow performance
Muscle recruitment	Normal recruitment of prime movers only, or minimal recruitment of accessory and stabilizing muscles of the trunk, neck or joints stabilizers	Some recruitment of accessory and stabilizing muscles of the trunk, neck or joints stabilizers	Pronounced recruitment of accessory and stabilizing muscles of the trunk, neck or joints
Reaction of the autonomic nervous system	Minimal increase in heart rate	Moderate increase in heart rate and respiration	Substantial increase in heart rate, respiration rate and significant sweating

The level of physical effort during non-material handling tests was determined on the basis of observational criteria indicative of no or slight limitation/problem, some functional limitation/problem, or significant limitation/problem [28]. Maximal effort was assumed when, on the basis of the expertise of the functional capacity evaluation (FCE) rater, sufficient criteria indicative of substantial functional problem/limitation were observed. Submaximal effort was assumed when a participant stopped a non-material handling test before the FCE rater observed sufficient criteria of substantial functional problem/limitation. Appendix 2 is used with permission from Verein IG Ergonomie, Swiss Association of Rehabilitation

^a ^Asymmetry (unequal loading) or deviation from neutral

## Abbreviations

FCE: Functional capacity evaluations
P_ED_: Physical effort determination by categorical observational criteria
S_ED_: Submaximal effort determination by dichotomous rating
CMP: Chronic nonspecific musculoskeletal pain
